# Important differences and meaningful changes for the Functional Assessment of Cancer Therapy-Cognitive Function (FACT-Cog)

**DOI:** 10.1186/s41687-018-0071-4

**Published:** 2018-10-12

**Authors:** M L Bell, H M Dhillon, V J Bray, J L Vardy

**Affiliations:** 1 Department of Epidemiology and Biostatistics, Mel and Enid Zuckerman College of Public Health, University of Arizona, 1295 N Martin Ave, Tucson, AZ 85724 USA; 2 Psycho-Oncology Co-operative Research Group, School of Psychology, University of Sydney, Sydney, NSW Australia; 3 Centre for Medical Psychology & Evidence-based Decision-making, School of Psychology, University of Sydney, Sydney, NSW Australia; 4 Department of Medical Oncology, Liverpool Hospital and University of Sydney, Sydney, NSW Australia; 5 Concord Cancer Centre and Sydney Medical School, University of Sydney, Sydney, NSW Australia

**Keywords:** Oncology, Cancer, Chemotherapy, Cognitive impairment, Quality of life, Minimal clinically important difference, FACT-Cog, Responder, Clinically important difference, Cumulative distribution function

## Abstract

**Background:**

We estimated clinically important, group-level differences in self-reported cognitive function for the Functional Assessment of Cancer Therapy-Cognitive Function (FACT-Cog) instrument. We also investigated individual level change that could be considered meaningful for cancer survivors affected by cognitive impairment following chemotherapy, and that could be used for responder analyses. We used data from a multi-site randomized controlled trial in 242 participants that evaluated a web-based intervention for improving self-reported cognitive functioning in adult cancer survivors who reported cognitive impairment and who had adjuvant chemotherapy in the previous 6–60 months. We used anchor and distribution methods to estimate a range of clinically important differences (CIDs) and investigated meaningful change thresholds (MCTs) for the FACT-Cog and the Perceived Cognitive Impairments (PCI) subscale, post-intervention and at six-month follow-up with empirical cumulative distribution functions. Our primary anchor was the patient reported cognitive function subscale of the European Organization for Research and Treatment of Cancer Quality of Life-Cognitive Functioning Scale (EORTC-CF).

**Results:**

Most participants were female (95%) breast cancer survivors (89%). Correlation of changes in the FACT-Cog and the EORTC-CF were 0.55 post-intervention and 0.61 at follow-up. Anchor-based CID estimates for the FACT-Cog using our primary anchor were 11.3 points (post) and 8.8 (follow-up), which corresponds to a standardized effect size of 0.49 and 0.38; 8.6% and 6.6% of the total scale’s range. Anchor-based CID estimates for the FACT-Cog PCI subscale were 7.4 (post) and 4.6 points (follow-up), which corresponds to a standardized effect size of 0.50 and 0.31; 10.3% and 6.4% of the PCI range). Empirical cumulative distribution functions of change in FACT-Cog demonstrating possible MCTs showed that anchor change of none, minimally better and much better were well separated.

**Conclusions:**

The CID and MCT estimates from this manuscript can help in the design, analysis and interpretation of self-reported cognitive function in cancer patients and survivors.

## Background

Cognitive impairment related to cancer and cancer treatment is a major concern for many cancer survivors [1, 2]. Self-reported questionnaires and neuropsychological tests are the two main methods used to assess cognitive function, although the majority of studies have found a poor association between the two [2]. The etiology of cancer-related cognitive impairment remains unknown and there is a lack of evidence to guide how best to treat it. Further research and randomized controlled trials (RCTs) assessing interventions for cognitive impairment are urgently required.

The design, analysis and interpretation of studies that use patient reported outcomes (PROs), such as self-reported cognitive function in cancer survivors, can be enhanced by the identification of important differences between groups. This has been known as the minimum important difference (MID), or more recently, the clinical important difference (CID) [3].Important changes for individuals are called meaningful change thresholds (MCTs).

Historically, PRO researchers have referred to the MCT as the MID, however, with the 2009 FDA’s PRO Guidance for Industry’s shift from this terminology [4], and the recognition that the MID (which we will refer to henceforth as the CID) was being estimated not on differences between groups, but on changes within group, the thinking about CIDs and MCTs has evolved. Although the terminology is changing [3], there is considerable overlap in the methodology for investigating CIDs and MCTs, as described below.

CIDs (i.e., differences between groups, such as treatment arms in a clinical trial) are important in study design in order to calculate power and sample size. MCTs are useful in interpretation of results for individuals and can be used to define a “responder” for responder analyses [4, 5]. MCTs are larger than CIDs because there is more uncertainty around individuals than groups (similar to why prediction intervals, where inference is on individuals, are always wider than confidence intervals, where inference is on groups). Best practice for discovering MCTs is still developing. For further review and emerging methods, see [3, 5–8].

Both the MCT and the CID can vary with patient population and setting, and as Revicki et al., state, “Confidence in a specific MID value evolves over time and is confirmed by additional research evidence” [9]. Further, there is growing recognition that both MCT and CID may best be described as a range, rather than a single number [10]. There are two approaches for investigating CIDs and MCTs. The primary approach is to link changes in the PRO to an anchor, which can be clinical (e.g., disease progression), or patient ratings of changes in health [9]. A second method, which can be used to provide supporting evidence for anchor based estimates, are distribution based methods [9]. Distribution based methods use the between-participant standard deviation to characterize changes. Details of both approaches are given in the Methods section.

One approach for investigating important changes in individuals, endorsed by the FDA, is to display the entire distribution of changes for individuals, the empirical cumulative distribution function (ECDF) [4, 5, 7, 11]. This approach is congruent with the idea of not choosing a single value for an MCT. When investigating MCTs, the ECDFs should be stratified by categorized anchor values (e.g., much worse, minimum worse, same, minimum better, much better); when considering trial results, stratification should be by trial arm (e.g., intervention, control). An effective intervention would have large separation between the curves.

Cheung and colleagues carried out a study to identify the CID for a cognitive function assessment instrument, the Functional Assessment of Cancer Therapy Cognitive Function (FACT-Cog), [12] using 220 breast cancer patients from Singapore whose cognitive functioning mostly declined [13]. To our knowledge, this was the first assessment of CID for this instrument. Our team undertook a randomized controlled trial (RCT) using the FACT-Cog, where many participants reported improvement in cognitive functioning [14]. CIDs and MCTs can differ based on the direction of change (i.e., deterioration or improvement), thus it is important to estimate them in both settings [9, 15]. Furthermore, the authors did not score the questionnaire using the recommended rubric (described below). Thus, we identified an opportunity to add to the body of knowledge surrounding this important patient reported outcome. Our trial and others we have designed, used the “Perceived Cognitive Impairments” (PCI) subscale from the FACT-Cog as its items appear to align well with our patients’ and study participants’ cognitive concerns. Thus, we aimed to estimate the FACT-Cog CID for the total score and the PCI subscale using data from 242 cancer survivors in an RCT set in Australia. We also aimed to investigate MCTs for this PRO in this population. Finally we aimed to show methods for investigating both CIDs and MCTs to help distinguish the two concepts.

## Methods

We used anchor and distribution methods to estimate a range of CIDs and MCTs for the FACT-Cog (total score) and the FACT-Cog PCI subscale. We used two anchors: the patient reported cognitive function subscale of the European Organization for Research and Treatment of Cancer-Quality of Life Questionnaire-Core 30 (EORTC-QLQ-C30-CF) as the primary anchor, and the Functional Assessment of Cancer Therapy-General (FACT-G), as a secondary anchor. These measures are described below.

### Study setting

We carried out an RCT at 18 Australian sites evaluating an intervention for improving self-reported cognitive functioning in adult cancer survivors. Eligible participants were at least 18 years old with any solid primary tumor (excluding malignancies of the central nervous system), who reported sustained cognitive symptoms after three or more cycles of adjuvant chemotherapy received in the previous 6–60 months [14].The intervention consisted of a 15-week home-based, web-based cognitive training program “Insight” versus usual care [16]. The primary outcome was the FACT-Cog perceived cognitive impairment subscale. Participants were measured at baseline (T1), post-intervention (T2), and 6 months post-intervention (T3). Primary trial results can be found elsewhere [14]. Ethical approval and consent were obtained for the original study.

### Measures

#### FACT-Cog

The Functional Assessment of Cancer Therapy Cognitive Function version 3 (FACT-Cog) FACT-Cog is a 37-item member of the FACIT suite of questionnaires (Functional Assessment of Chronic Illness Therapy) [17]. The FACT-COG is made up of four subscales: perceived cognitive impairments (PCI; 18 items); perceived cognitive abilities (7 items); impact of perceived cognitive impairment on QOL (4 items); and comments from others on cognitive function (4 items). The FACT-Cog has been found to be reliable and valid, and has been used in various cancer populations [12]. The FACIT’s recommended scoring method is to use 33 items and to score the four subscales separately. (For scoring instructions, see www.FACIT.org.) We investigated CIDs and MCTs for the PCI subscale, as well as a total score, derived by summing the recommended 33 items. The response options range from 0 to 4. Negative items were reverse scored for the total scores, so that higher values indicate better self-reported cognitive functioning. Cheung et al. used all 37 items, so we also computed a total score this way, in order to compare results. The possible ranges are 0–72, 0–132 and 0–148 for the PCI subscale, 33- and 37- item totals respectively.

#### EORTC-QLQ-C30

The European Organization for Research and Treatment of Cancer Quality of Life Questionnaire Core 30 (EORTC-QLQ-C30) is a widely used 30-item instrument for assessing quality of life in cancer patients [18]. It has been shown to be reliable and valid [19]. We focused on the two items which make up the cognitive functioning scale, EORTC-CF: “Have you had difficulty in concentrating on things, like reading a newspaper or watching television?” and “Have you had difficulty remembering things?”. The EORTC-CF is scored by using a transformation from the 4-point Likert scale to a 0–100 point scale, with higher values indicating better cognitive functioning, and with possible values: 0, 16.67, 33.34, 50, 66.67, 83.33, 100 [18, 20].

#### FACT-G

The Functional Assessment of Cancer Therapy-General (FACT-G) is a reliable, valid, commonly-used questionnaire for assessing health-related quality of life in cancer populations. [21]. There are 27 items and four subscales: physical, social, emotional and functional well-being. The possible range is 0–108, with higher values corresponding to greater quality of life. We used this measure because its CID has been investigated in a variety of populations [17], but, since it does not directly assess cognitive functioning, we used it as a secondary anchor.

### Statistical methods

Descriptive statistics of demographics were computed. Means and standard deviations (SDs) were computed for each of the subscales and the total score, at each of the time points, by treatment arm. Cronbach’s alpha was calculated for the FACT-Cog items at each time point to assess internal consistency. Change from baseline was calculated by subtracting the baseline score from each of the post-baseline scores, so that positive values indicate improvement in cognitive functioning. All analyses used SAS version 9.4 (Cary, North Carolina).

### Anchor based assessment of CIDs

Polyserial correlation (appropriate for ordinal variables with continuous variables) on the change in FACT-Cog and the categorized anchor changes was computed. Minimum correlation magnitude ranging from 0.30–0.37 has been recommended for anchors [22, 23], so we used these values as benchmarks. The range of the EORTC-CF is 0–100, so the change from baseline in the EORTC-CF can range from − 100 to 100, with increments of 16.67 [20]. Thus, as in Cheung et al., we categorized each change from baseline as much worse (< − 16.67), minimally worse (− 16.67), no change (0), minimally better (16.67), and much better (> 16.67). I.e., a one-category change is minimally better and 2 or more is much better, etc. The value for improvement (16.67) corresponds to an improvement by one category on one of the items on the EORTC-CF and a stable response on the other. The mean change in FACT-Cog was computed for each of these categories. The CID was estimated as the difference in scores for minimally better and no change [11], which follows recommendations that using larger change may overestimate the CID [9], and follows the methods of Cheung et al. A change by one category is likely to be meaningful, as there are only four categories in the EORTC-CF. We did not investigate deterioration because so few participants declined (*n* = 11 at T2, *n* = 7 at T3).

Standardized effect sizes (ES) were estimated by dividing the CID estimate by the overall baseline standard deviation (SD). Similar methods were used for the secondary anchor FACT-G, with categorizations of much worse (< − 7 points), minimally worse (− 7 to − 4 points), no change (− 3 to 3 points), minimally better (4–7 points) and much better (> 7 points). These values were arrived at by examining MID estimates of the FACT-G [17].

### Distribution based assessment of the CID and the MCT

Distribution based methods for the CID and MCT have been recommended to support evidence gained from anchor-based methods [9, 24]. Recommended CID estimates include calculating one half and one third the SD of the score. The standard error of measurement (SEM) has been considered as a lower bound for the MCT [5]. We used the SD at each time point. The SEM was calculated using SEM = *σ*√(1 − *ρ*_*T-RT*_), where *ρ*_*T-RT*_ = test-retest reliability [25]. We used the values *ρ*_*T-RT*_ = 0.74, obtained from a FACT-Cog validation study [26], in addition to the control group’s correlation between baseline and post-intervention *ρ*_*T-RT*_ = 0.70. (This was the value for both the FACT-Cog total score and the PCI subscale.) The control arm was used for estimating test-retest reliability because they would presumably experience less change than the intervention arm, thereby better approximating reliability. Both arms were used for all other estimation.

### Anchor based investigation of the MCT

We graphed one minus the cumulative distribution function of the change from baseline in the FACT-Cog stratified by time (T2 and T3) and categories of the EORTC-CF anchor change of “no change”, “minimally better”, and “much better”, as defined above. Specifically, we graphed the proportion of participants, F(x) = 1 - CDF, whose change from baseline was greater than or equal to x. As described above this was not done for deterioration, due to the small numbers. We plotted vertical lines at various thresholds to indicate possible MCTs, and showed the proportion of responders using these possible cutoffs in each of the three anchor categories. These thresholds were averaged over T2 and T3 and were the rounded values of: 1) the standard error of measurement (for *ρ*_*T-RT*_ = 0.70) and 2) the difference in the mean value of the change in FACT-Cog for those in the anchor category of “much better” and “same”. A reference line of no change was also shown.

### Sensitivity analysis

As missing data in PROs can bias estimates, we performed sensitivity analyses [27–29]. Multiple imputation (MI) was undertaken and EORTC anchor based CID estimates for the FACT-Cog total score were computed. Imputation models included the FACT-Cog total score, the EORTC-CF score, previous cognitive problems (see Table 1), age, treatment arm, and other PROs (stress, health-related quality of life, and anxiety). Imputation was performed in “wide form”, with one record per subject (in contrast to “long form”, with a record per subject, per assessment time), so that within-subject correlation was maintained. Twenty-five imputed datasets were created using the Markov Chain Monte Carlo method, as implemented in SAS version 9.4. CIDs were estimated for each of the 25 complete datasets [30] using anchor and distribution methods as described above, and averaged, following Rubin’s rules [31].

**Table 1:** Participant characteristics of a randomized controlled trial evaluating a web-based intervention for improving self-reported cognitive functioning in adult cancer survivors

	Intervention *N* = 121	Control *N* = 121	Combined *N* = 242
Female	116 (96%)	114 (94%)	230 (95%)
Age (range); years	52 (23–74)	54 (31–74)	53 (23–74)
Median education (range); years	14 (8–19)	12 (3–19)	13 (8–19)
English as first language	117 (97%)	117 (97%)	234 (97%)
Primary tumor type			
Breast	108 (89%)	108 (89%)	216 (89%)
Colorectal	6 (5%)	7 (6%)	13 (5%)
Other	7 (6%)	6 (5%)	13 (5%)
Time since completion of chemotherapy, months, mean (range)	27 (6–57)	27 (6–60)	27 (6–60)
Radiotherapy	86 (71%)	78 (64%)	164 (68%)
Immune therapy	30 (25%)	24 (20%)	54 (22%)
Hormone therapy	84 (69%)	85 (70%)	171 (71%)
Previous neurological history^a^	20 (17%)	30 (25%)	50 (21%)

^a^Defined as: held back a grade in school; required remedial help at school; diagnosed with a learning disability; head injury with loss of consciousness with residual sequelae; history of seizures, dementia, coma, epilepsy, cardiac arrest requiring cardiopulmonary resuscitation, stroke; history of other neurological risk; or history of significant alcohol abuse

## Results

We report the results for the 33-item FACT-Cog total score in this manuscript. Results for the 37-item total score are given in the Appendix. Cronbach’s alpha at each time point was estimated as 0.89, 0.89, and 0.89 for the FACT-Cog at baseline, post-intervention and 6 months respectively; 0.94, 0.96, 0.96 for the FACT-Cog PCI subscale; 0.29, 0.73 and 0.69 for the EORTC-CF; and 0.60, 0.63, and 0.70 for the FACT-G.

### Patient population

Table 1 shows participant characteristics. The majority of the participants were female (230, 95%) breast cancer survivors (216, 89%). The median age was 53 years (range 23–74) and the mean time since completion of chemotherapy was 27 months (range 6–60). Table 2 gives means and SDs at each time point for each of the instruments and subscales. At T2 there were 192/242 (79%) complete FACT-Cog scores and at T3 there were 184/242 (76%).

**Table 2:** Means and SDs for each of the FACT-Cog subscales and total scores, at each of the time points, by treatment arm

Instrument/ Subscale^a^	T1: Baseline		T2: Post-intervention		T3: Six-months	
	Intervention *N* = 121	Control *N* = 121	Intervention *N* = 94	Control *N* = 98	Intervention *N* = 95	Control *N* = 89
EORTC-CF	41.5 (8.8)	39.1 (16.9)	67.9 (22.8)	58.3(19.2)	68.6 (22.4)	60.4 (20.6)
FACT-G	76.5 (15.3)	77.1 (14.1)	83.5 (14.0)	82.1 (13.3)	84.2 (14.4)	80.2 (14.2)
FACT-Cog-PCI	38.6 (14.3)	41.9 (15.1)	23.2 (15.5)	33.5(16.1)	23.4 (14.6)	32.6 (16.5)
FACT-Cog-QoL	7.5 (4.2)	7.7 (4.4)	4.2 (4.3)	5.7 (4.2)	4.3 (4.3)	5.7 (4.3)
FACT-Cog-Oth	3.0 (3.6)	3.3 (3.7)	1.5 (2.0)	2.5 (2.79)	1.8 (3.1)	2.5 (2.8)
FACT-Cog-PCA	12.0 (5.0)	12.5 (5.6)	17.2 (5.8)	14.1 (5.5)	16.9 (6.0)	14.1 (5.5)
FACT-Cog-Total (33 items)	78.8 (22.5)	75.6 (23.7)	104.3 (25.1)	88.3 (25.2)	103.5 (25.0)	89.3(25.8)
FACT-Cog-Total (37 items)	85.6 (25.5)	81.5 (26.7)	114.2 (27.7)	95.6 (28.7)	113.4 (28.6)	97.0 (29.2)

^a^*Cog-PCI* Perceived cognitive impairment (18 items), *Cog-QoL* Impact of perceived cognitive impairment on QOL (4 items), *Cog-Oth* Comments from others on cognitive function (4 items), *Cog-PCA* Perceived cognitive abilities (7 items)

### Total score CID estimation

The polyserial correlation between the categorized change in EORTC-CF and the change in the FACT-Cog was 0.55 for T2 and 0.61 for T3. Estimates were well over the benchmark of 0.3–0.37, indicating that the EORTC-CF was a reasonable anchor. The correlation between the categorized change in the secondary anchor, the FACT-G, and the change in the FACT-Cog was 0.44 for T2 and 0.47for T3. While this correlation is reasonable, the lower value in comparison to the EORTC-CF indicates that more confidence should be placed on the primary anchor (in addition to the EORTC-CF measuring the same concept, cognition).

CID estimates for the 33-item total score were 11.3 and 8.8 for the primary anchor at T2 and T3 (Table 3). These values correspond to standardized effect sizes of approximately 0.5 and 0.4 SD, and 8.6 and 6.6% of the possible range of the scale. The average of these two values is 10.0 (standardized effect size 0.42, 7.6% of the total scale). CID estimates using the FACT-G were 9.9 (T2) and 6.1 (T3). See Appendix. Estimates using distribution criteria are shown in Table 4. Estimates range from 7.7 (1/3 SD at baseline) to 13.2 (1/2 SD at T2). Sensitivity analysis using multiply imputed data gave CID estimates of 10.8 at T2, and 8.1 at T3, which are similar to the primary results. Lower limits of the MCT using the SEM ranged from 11.8 to 14.4.

**Table 3:** Anchor-based MCT estimates for improvement for the FACT-Cog total score (33 item) and FACT-Cog-PCI based on changes from baseline at post-intervention (T2) and six month follow-up (T3) using the primary anchor EORTC-CF. Results from sensitivity analysis shown in parenthesis in the last three columns^a^. Correlations, ρ, are for the change in the FACT-Cog total score or PCI subscale and the change in anchor (categorized)

FACT-Cog scale	Time	Anchor change^b^	N (%)	Mean change in FACT-Cog (SD)	CID Estimate^c^	Standardized effect size	% of scale
Total score	T2	ρ = 0.55					
		Much better	92 (48)	27.8 (22.1)			
		Minimally better	50 (26)	15.1 (15.3)			
		No change	39 (20)	3.8 (13.9)	11.3 (10.8)	0.49 (0.47)	8.6 (8.0)
		Minimally worse	10 (5)	5 (15.6)			
		Much worse	1 (0.5)	12 (−)			
	T3	ρ = 0.61					
		Much better	85 (47)	29.1 (20.7)			
		Minimally better	56 (31)	15.8 (15.1)			
		No change	35 (19)	7.1 (14.6)	8.8 (8.1)	0.38 (0.36)	6.6 (6.1)
		Minimally worse	6 (3)	−14.7 (14.0)			
		Much worse	1 (0.6)	7 (−)			
FACT-Cog PCI	T2	ρ = −0.49					
		Much better	92 (48)	−16.9 (13.7)			
		Minimally better	50 (26)	−9.8 (9.9)			
		No change	39 (20)	−2.4 (9.8)	7.4 (6.9)	0.50 (0.47)	10.3 (9.7)
		Minimally worse	10	−4.8 (11.8)			
		Much worse	1	−8.0 (−)			
	T3	ρ = −0.54					
		Much better	85 (47)	−17.9 (12.7)			
		Minimally better	56 (31)	−10.1 (10.1)			
		No change	35 (19)	−5.5 (9.6)	4.6 (6.5)	0.31 (0.44)	6.4 (9.1)
		Minimally worse	6 (3)	4.8 (8.7)			
		Much worse	1 (0.6)	−4.0 (−)			

^a^Sensitivity analysis using 25 multiple imputed datasets

^b^Change in EORTC-CF < −16.67 (much worse), −16.67 (minimally worse), 0 (no change), 16.67(minimally better), and > 16.67 (much better)

^c^|Anchor min better – anchor no change| in mean change in FACT-Cog

**Table 4:** Distribution-based CID (SD multiples) and MCT lower limit (SEM) estimates for the FACT-Cog total score and perceived cognitive impairments subscale

	N	SD	One-third SD	One-half SD	1 SEM, *ρ*_*T-RT*_ = 0.74	1 SEM, *ρ*_*T-RT*_ = 0.70
FACT-Cog score (33 items)						
T1: Baseline	242	23.13	7.7	11.6	11.8	12.7
T2: Post-intervention	192	26.31	8.8	13.2	13.4	14.4
T3: Six-month follow-up	184	26.29	8.8	13.1	13.4	14.4
Mean		25.24	8.4	12.6	12.9	13.8
FACT-Cog PCI						
T1: Baseline	242	14.77	4.9	7.7	7.5	8.1
T2: Post-intervention	192	16.61	5.5	8.8	8.4	9.1
T3: Six-month follow-up	184	16.17	5.4	8.8	8.2	8.9
Mean		15.85	5.3	7.9	8.1	8.7

Definitions: *SD* Standard deviation, *SEM* Standard error of measurement = *σ*√(1 − *ρ*_*T-RT*_)

The CID estimates for the 37-item FACT-Cog total score were 12.0 and 10.4 using the primary anchor, at T2 and T3 respectively (standardized effect size = 0.46 and 0.40; 9.1 and 7.0% of the possible range of the total score). Using distribution methods (SD multiples), CID estimates ranged from 8.7 to 15.0. See Appendix.

### Perceived cognitive impairments subscale CID

Change over time for the PCI subscale score correlated well with the change in categorized EORTC-CF; the correlations were .0–.49 and − 0.54, indicating that the anchor was reasonable. Similar to the total score, the magnitude of the correlation with the change in the secondary anchor, FACT-G, was lower, at − 0.34 and − 0.38. Primary anchor based CID estimates for the PCI subscale were 7.4 (T2) and 4.6 (T3), (0.5 and 0.3 SD, 10.3 and 6.4% of the subscale). Sensitivity estimates were similar. Distribution based estimates ranged from 4.9 to 8.8. See Tables 3 and 4.

### Investigation into MCTs

**Fig. 1:**
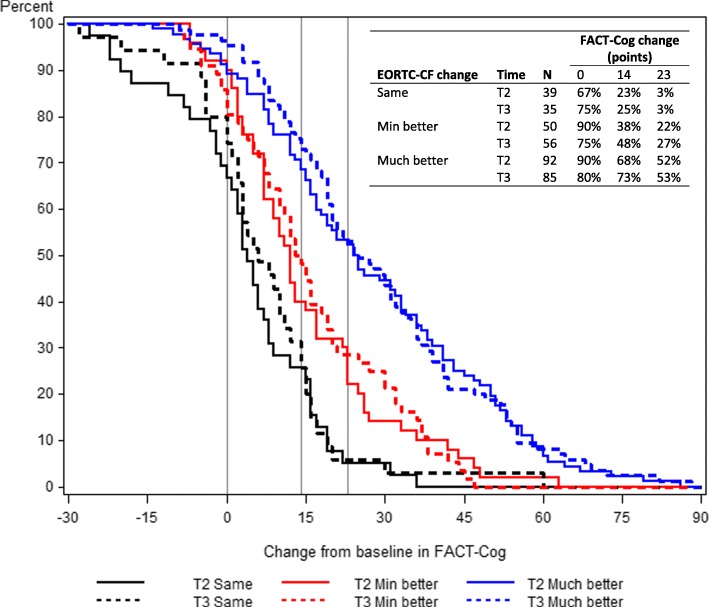
Empirical cumulative distribution functions of change from baseline in the FACT-Cog, stratified by categorized anchor change (EORTC-CF) and time (T2 = post intervention, T3 = 6 months). Larger values of change are better. Possible meaningful change thresholds of 14 (SEM) and 23 points (difference between “much better” and “same”) are shown as vertical lines, as well as change of 0. The table gives percentages of participants who changed by at least a given amount

Figure 1 shows the empirical CDF curves for the change in FACT-Cog stratified by time and the EORTC-CF anchor changes of none, minimally better and much better. These curves show a range of possible MCTs (vertical lines) that could be used for defining responders, as well as no change for reference. These thresholds were rounded averages over T2 and T3 and were: 14 = standard error of measurement (for *ρ*_*T-RT*_ = 0.70); and 23 = the difference in the mean value of the change in FACT-Cog for those in the anchor category of “much better” and “same”. The curves between categories are well-separated, and the curves within categories are similar. The table within the graph shows the percentage of participants who changed by at least a given amountFor example, changes of at least 14 points were experienced by 23% (T2) and 25% (T3) of participants in the “same” anchor category; 38% (T2) and 48% (T3) in the “min better” category; and 68% (T2) and 73% (T3) in the “much better” category.

## Discussion

We estimated clinically important differences in the 33-item FACT-Cog total score and the perceived cognitive impairments subscale using data from 242 cancer survivors, in an RCT that evaluated a web-based cognitive training program. Estimates for the total score ranged from 6.1 to 13.2 points. The mean of the T2 and T3 anchor-based estimate using our primary anchor, the EORTC-CF, was 10.0 points, corresponding to a standardized effect size of 0.42, which is 7.6% of the total scale. CID estimates for the FACT-Cog PCI subscale ranged from 3.1 to 8.8. The mean of the T2 and T3 anchor-based estimate using our primary anchor was 5.9 points, corresponding to a standardized effect size of 0.40, which is 8.4% of the PCI subscale total.

The standardized effect sizes of our anchor based CID estimates ranged from about 0.21 to 0.47. This is consistent with many other estimated PRO CIDs [32], and corresponds to a small (0.2) to medium (0.5) effect size [33]. We aimed to compare our estimates to Cheung et al. by following their methods and using the same anchor to increase comparability. They estimated the CID for deterioration in the 37-item total score at 9.6 points, with all estimates ranging from 6.9 to 10.6 points (4.7–7.2% of the total score). Our CID estimate for improvement in the 37-item score ranged from 8.7 to 15.0 (5.9–10.1% of the total score). Our study adds considerably to their findings, as only 13% of their participants reported improvement, as compared to 46% in our study.

When designing studies, researchers should undertake power and sample size calculations that use a target difference that is both important and realistic [34]. In this paper we estimated CIDs, which address the issue of importance. Pilot studies, review of evidence and seeking the opinions of experts and stakeholders can give an idea about whether the specified difference is realistic [34].The range of estimates for the CID can also be used to perform sensitivity analyses for their sample size calculations, as demonstrated in reference [35].

We also explored individual change thresholds using cumulative distribution functions. The largest MCT candidate, 23 points change, separated the anchor change categories well and was consistent between time points. The value of 14 was not as consistent between time points and did not show as much separation between categories. However, any choice of MCT to define a responder must consider false positives (more likely with lower MCT) and false negatives (more likely with a higher MCT).

The CID estimates were consistently smaller for T3 (six-month follow-up) as compared to T2 (post-intervention, approximately 15 weeks), which can also be seen in the CDF curves. There is no clear reason for this. The response rate was similar between the two time points. Researchers should consider what the most appropriate CID is for their study based on their primary outcome and its timing.

### Study limitations

A limitation of this study was that due to the nature of the intervention the original RCT was not blinded, and this may have affected patients’ self–reported assessments of their cognition [36]. Also, a clinical anchor that is simple and easy to interpret would be ideal [5], but this was not possible as there were no clinical assessments in this internet-based study. We believe that while the 2-item EORTC-CF score is not immediately interpretable, the mapping of the possible change scores was reasonable, has been used before [13] and had correlation well above the benchmark for anchors. Furthermore, we included a second anchor, the FACT-G, which is widely used and has been well studied with regard to important change estimation. The EORTC-CF had low internal consistency at baseline, with Cronbach’s alpha = 0.29, but it increased dramatically to 0.73 and 0.69 at the follow-up times when CIDs and MCTs are assessed. Low alpha values can occur when there are few items and/or when an item’s observed range is small, and no one at baseline responded that they had no difficulty remembering things (item 2 of the EORTC-CF). The FACT-G also had relatively low Cronbach’s alpha, despite it having 27 items. This may be due to the heterogeneity of the sample due to variation in time since chemotherapy, which ranged from 6 to 60 months. Our sample was comprised of mostly breast cancer survivors, it is possible that important differences and changes vary by cancer type and/or sex. A strength of this study included estimating the CID using several methods, including a sensitivity analysis using multiple imputation. A further strength is the high correlation between the primary anchor and the FACT-Cog.

It is useful to investigate both differences and changes for the same measure, so that readers can distinguish estimation methods and uses for both. Important differences for groups, can be used to design studies, by powering on the CID. ECDFs and MCTs are useful in interpretation of how interventions affect individuals.

## Conclusions

Measuring perceived cognitive complaints is an important component of assessing cognition and symptoms in cancer patients, and can give oncologists guidance for determining the proportion of patients who improve over time for a given treatment. By estimating and reporting CIDs and MCTs our study provides information for the design, analysis and interpretation of studies using the FACT-Cog to evaluate self-reported cognitive function in cancer patients and survivors.

## Appendix

**Table 5:** Anchor-based CID estimates for improvement for the FACT-Cog total score (33 item) and FACT-Cog-PCI based on changes from baseline at post-intervention (T2) and six month follow-up (T3) and **secondary anchor FACT-G**. Correlations, ρ, are for the change in the FACT-Cog total score or PCI subscale and the categorized anchor changes

FACT-Cog scale	Time, Anchor	Anchor change^a^	N (%)	Mean change in FACT-Cog (SD)	MCT Estimate^b^	Standardized effect size	% of scale
Total score	T2, FACT-G	ρ = 0.44					
		Much better	73 (38)	28.7 (22.5)			
		Minimally better	27 (14)	18.5 (21.1)	9.9 (9.1)	0.43 (0.40)	7.5 (6.9)
		No change	43 (22)	8.6 (12.4)			
		Minimally worse	26 (14)	6.9 (17.0)			
		Much worse	13 (7)	12.9 (17.2)			
	T3, FACT-G	ρ = 0 0.47					
		Much better	57 (31)	30.7 (22.6)			
		Minimally better	35 (19)	21.2 (18.6)	6.1 (5.5)	0.42 (0.24)	4.7 (4.1)
		No change	38 (21)	15.1 (14.5)			
		Minimally worse	22 (13)	10.8 (18.0)			
		Much worse	20 (12)	5.6 (20.4)			
FACT-Cog PCI	T2, FACT-G	ρ = −0.34					
		Much better	73 (38)	−16.5 (14.8)			
		Minimally better	27 (14)	−11.0 (14.5)			
		No change	43 (22)	−5.3 (10.6)	6.3 (4.4)	0.32 (0.30)	6.6 (6.1)
		Minimally worse	26 (14)	−10.5 (10.6)			
		Much worse	13 (7)	−18.0 (14.3)			
	T3, FACT-G	ρ = −0.38					
		Much better	57 (31)	−18.0 (14.4)			
		Minimally better	35 (19)	−13.5 (11.7)			
		No change	38 (21)	−10.4 (9.1)	3.1 (3.1)	0.21 (0.21)	4.4 (4.4)
		Minimally worse	22 (13)	−8.3 (12.1)			
		Much worse	20 (12)	−5.1 (12.0)			

^a^Change in FACT-G < −7 (much worse), −7 to −4 (minimally worse), −3 to 3 (no change), 4 to 7 (minimally better), and > 7 (much better)

^b^|Anchor min better – anchor no change| in mean change in FACT-Cog

**Table 6:** Anchor-based CID estimates based on changes from post-intervention and six month follow-up for the **37-item** FACT-Cog total score

EORTC change	N (%)	Mean change in FACT-Cog (SD)	CID Estimate^a^	Standardized effect size (% of scale)
T2:ρ = 0.58				
Much better	92 (48)	31.3 (25.2)		
Minimally better	50 (26)	16.5 (16.7)		
No change	39 (20)	4.5 (15.9)	12.0	0.46 (9.1)
Minimally worse	10 (5)	4.6 (18.1)		
Much worse	1 (0.5)	15 (−)		
T3:ρ = 0.63				
Much better	85 (47)	33.0 (23.8)		
Minimally better	56 (31)	17.9 (17.0)		
No change	35 (19)	7.5 (16.2)	10.4	0.40 (7.0)
Minimally worse	6 (3)	−16.7(16.1)		
Much worse	1 (0.6)	10 (−)		

^a^|Min better – No change| in mean change in FACT-Cog

**Table 7:** Distribution-based CID (SD multiples) and MCT lower limit (SEM) estimates for the 37-item FACT-Cog total score based on changes from post-intervention and six month follow-up

FACT-Cog score (37 items)	SD	One-third SD	One-half SD	1 SEM, ρ = 0.74	1 SEM, ρ = 0.70
T1: Baseline	26.13	8.7	13.1	13.3	14.3
T2: Post-intervention	30.10	10.0	15.0	15.3	16.5
T3: Six-month follow-up	30.00	10.0	15.0	15.3	16.4
Mean	28.73	9.6	14.4	14.7	15.7

Definitions: *SD* Standard deviation, *SEM* Standard error of measurement = *σ*√(1 − *ρ*_*T-RT*_)

## Abbreviations

CI: Confidence interval
CID: Clinical important difference
EORTC-CF: European Organization for Research and Treatment of Cancer Quality of Life-Cognitive Functioning Scale
FACT-Cog: Functional Assessment of Cancer Therapy Cognitive Function
FACT-G: Functional Assessment of Cancer Therapy General
MCT: Meaningful change threshold
MID: Minimal important difference
PRO: Patient reported outcomes
RCT: Randomized controlled trial
SD: Standard deviation
SEM: Standard error of measurement
